# Ecophenotypic plasticity leads to extraordinary gastropod shells found on the “Roof of the World”

**DOI:** 10.1002/ece3.1586

**Published:** 2015-07-03

**Authors:** Catharina Clewing, Frank Riedel, Thomas Wilke, Christian Albrecht

**Affiliations:** 1 Department of Animal Ecology and Systematics, Justus Liebig University GiessenGiessen, Germany; 2 Palaeontology, Institute of Geological Sciences, Freie Universität BerlinBerlin, Germany; 3 Key Laboratory of Plateau Lake Ecology and Global Change, College of Tourism and Geography, Yunnan Normal UniversityYunnan, China

**Keywords:** Corkscrew-like, *Gyraulus*, Planorbidae, Steinheim Basin, Tibetan Plateau

## Abstract

The often extraordinary shell forms and shapes of gastropods found in palaeolakes, such as the highly diverse *Gyraulus* fauna of the famous Steinheim Basin, have been puzzling evolutionary biologists for centuries, and there is an ongoing debate whether these aberrant shell forms are indicative of true species (or subspecies) or ecophenotypic morphs. Interestingly, one of the Steinheim *Gyraulus* morphs – a corkscrew-like open-coiled shell – has a recent analogue in the Lake Bangong drainage system on the western Tibetan Plateau. Therefore, a combination of morphological, molecular, palaeolimnological, and ecological analyses was used in this study to assess whether the extraordinary shell shape in *Gyraulus* sp. from this drainage system represents a (young) ecophenotypic phenomenon or if it has been genetically fixed over an extended period of time. Our morphological, ecological, and palaeolimnological data suggest that the corkscrew-like specimens remain restricted to a small pond near Lake Bangong with an elevated pH value and that the colonization may have occurred recently. The phylogenetic reconstruction based on two gene fragments shows that these nonplanispiral specimens cluster within the previous described Tibetan Plateau *Gyraulus* clade N2. A network analysis indicates that some haplotypes are even shared by planispiral and nonplanispiral specimens. Given the ephemerality of the phenomenon, the compact network patterns inferred, the likely young phylogenetic age of the aberrant *Gyraulus* shells studied, and the ecological peculiarities of the study site, we suggest that the evolution of the aberrant shell forms on the Tibetan Plateau could likely be considered as a rapid ecophenotypic response, possibly induced by ecological stress. This finding may thus have implications for the ongoing debate about the processes that have caused the extraordinary shell diversity in palaeolakes such as the Steinheim Basin.

## Introduction

Molluscan shell shape diversity represents a prime character for the interpretation of, for example, faunal changes (Williamson 1981; Wagner and Erwin 2006) and ecological shifts (Geary et al. 2002; Albrecht et al. 2010) and prominently figures in discussions about speciation and extinction processes through time (e.g., Van Bocxlaer et al. 2008; Salzburger et al. 2014). The often high degree of plasticity of shell shape characters, however, may lead to challenges in evolutionary and palaeontological studies (e.g., Palmer 1985; Van Bocxlaer et al. 2008; Pfennig et al. 2010). It is therefore important to understand how shell shape changes over time and how it relates to environmental modulation. One famous fossil example is the gastropod fauna of the highly debated Miocene Steinheim Basin (southwestern Germany). This basin represents a long-lived palaeo lake and is well known for its rich and morphologically diverse shells of the freshwater gastropod genus *Gyraulus* (Pulmonata, Hygrophila, Planorbidae) (Hilgendorf 1867; Mensink 1984; Gorthner 1992; Nützel and Bandel 1993).

The typical *Gyraulus* shell is relatively small and planispiral (Meier-Brook 1983), a shape also found in the fossil record of the Steinheim Basin (as represented by the potential founder species of the flock, *G*. *kleini*; Rasser 2013). However, several aberrant shell forms have been observed such as large trochiform morphs with thick shells (e.g., *G*. *trochiformis*) and fragile nonplanispiral (open-coiled plus corkscrew-like = scalarid) forms (e.g., *G*. *denudatus*) (Nützel and Bandel 1993). Altogether, nineteen different forms have been described, and the relationship of which (illustrated by possible transitions) was summarized in the first ever fossil-based phylogenetic tree by Hilgendorf (1867). This example was also used to introduce Darwin’s theory of transmutation into palaeontology (Janz 1999). Hilgendorf's study started a long-lasting and ongoing discussion on fossil forms and the question whether they are either true species (or subspecies) or ecophenotypic morphs (for a detailed review, see Rasser 2013 and references therein). In this context, one significant but still largely unresolved question is which factors are ultimately responsible for the observed morphological “abnormalities” in the Steinheim gastropod fauna. It is generally assumed that abiotic factors such as, water chemistry and water current, and biotic factors, such as parasitism and predation, are potential triggers for shell-shape changes (e.g., Zuykov et al. 2012).

Extant species of the freshwater genus *Gyraulus* are well known for their rather large shell variability (Gorthner 1992). Deviations from the typical *Gyraulus* morphotype, representing potential analogues to some of the Steinheim morphs, were found, among others, in species inhabiting ancient lakes such as the Balkan lakes Ohrid and Prespa (e.g., *G*. *trapezoides* and *G*. *stankovici*, respectively; Hadžišče 1953; Hubendick and Radoman 1959), Lake Biwa (*G*. *biwaensis*; Meier-Brook 1983), and Lake Lugu (*G*. *luguhuensis*; Shu et al. 2013). In fact, aberrant shell forms, along with other biological and geological evidences, were even used as proxies to determine the ancient status of potential candidate lakes (Moore 1898; Wilke et al. 2007; Albrecht et al. 2009, 2012; Salzburger et al. 2014).

Interestingly, almost all of the remarkable morphological variations observed in the Steinheim *Gyraulus* fauna have been found in extant *Gyraulus* species except for the corkscrew-like open-coiled morph (see Table1). However, we recently found the latter shell form at a single location in the Bangong Co (Co = lake) drainage basin, which is located on the western Tibetan Plateau. The plateau, also known as the “Roof of the World”, is the highest (average elevation of about 5000 m a.s.l.) and largest (about 2.5 million km^2^) plateau on earth (Royden et al. 2008). It probably reached the current elevation during the Middle/Late Miocene (Royden et al. 2008; Spicer et al. 2003; Rowley and Currie 2006). Following controversial discussions about the extent of glaciation across the Tibetan Plateau during the Quaternary, it is nowadays generally accepted that the plateau was not completely covered by an extensive ice sheet (Derbyshire et al. 1991; Lehmkuhl and Owen 2005). Thus, freshwater organisms could have persisted in freshwater refugia (e.g., lakes, springs, streams) on the plateau during the last glacial maximum (LGM; about 20,000 years ago) or even longer as recently shown for the gastropod genus *Radix* spp. (Oheimb et al. 2011).

**Table 1:** Selected taxa of recent freshwater gastropods showing corkscrew-like shells

Taxon	Species	Location	Reference (plus shell shape)	
Caenogastropoda				
Amnicolidae	*Liobaicalia stiedae*^1^ (W. Dybowski, 1875)	Endemic to Lake Baikal, Russia	Sitnikova et al. (2001)	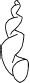
Cochliopidae	*Heleobia mirum*^1^ (Haas, 1957)	Endemic to Lake Titicaca, Bolivia/Peru	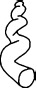	Kroll et al. (2012)
Hydrobiidae	*Gocea ohridana*^1^ (Hadžišce, 1956)	Endemic to Lake Ohrid, Macedonia/Albania	Hauffe et al. (2010)	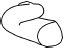
Heterobranchia				
Lymnaeidae	*Lymnaea stagnalis*^2^ (Linnaeus, 1758)	Former USSR, Germany, USA	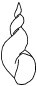	Zuykov et al. (2012)
Planorbidae	*Anisus leucostoma*^2^ (Millet, 1813)	Germany (laboratory)	Boettger (1949)	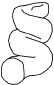
Planorbidae	*Gyraulus* sp.^2^	Lake Bangong system, Tibetan Plateau, China	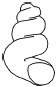	This study
Planorbidae	*Biomphalaria glabrata*^2^ (Say, 1818)	Saint Lucia (laboratory)	Basch (1968)	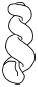
Valvatidae	*Valvata lewisi*^2^ (morph *ontariensis*) (F. C. Baker, 1931)	Lake Superior drainage, northwestern Ontario, Canada	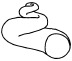	Burch (1989)

1 Corkscrew-like shell = species-specific character.

2 Corkscrew-like shell = intraspecific sporadical aberrant form.

In this study, we used a combination of morphological, molecular, palaeolimnological, and ecological data in order to assess whether the extraordinary shell shape in *Gyraulus* sp. from the Lake Bangong drainage system represents a (young) ecophenotypic phenomenon or if it has been genetically fixed over an extended period of time. As extensive genome and/or transcriptome studies were not feasible due to the limited material available from this extremely remote area and the material’s preservation status, the following specific tasks were carried out.

1. We first studied the phylogenetic status (including sister group relationships) of Tibetan Plateau *Gyraulus* spp. together with the phylogeographical status of Lake Bangong populations using mitochondrial DNA markers (a nonmonophyly of corkscrew-like specimens would possibly suggest a ecophenotypic phenomenon).
2. We then assessed the morphological variation of *Gyraulus* populations and its correlation with environmental variables based on shell and protoconch characteristics as well as on palaeolimnological and water chemistry information (a strong correlation between shell shape and (palaeo-)environment would indicate ecophenotypic processes).
3. Finally, we empirically assessed the temporal frame in which the evolution of the nonplanispiral shell shape has occurred (an “old” monophyletic group would possibly indicate genetic fixation).

In our study, we also discussed extraordinary shell forms (particularly corkscrew-like shells) in other extant freshwater gastropods in a comparative context relative to the underlying evolutionary processes. These insights might not only help ecologists and evolutionary biologists understanding the spatial and temporal patterns of shell shape evolution in extant freshwater systems (particularly ancient lakes), they may also shed light on the hotly debated processes leading to aberrant shell forms in palaeolakes such as in the famous Steinheim Basin.

## Material and Methods

### Study area and sampling

**Figure 1:**
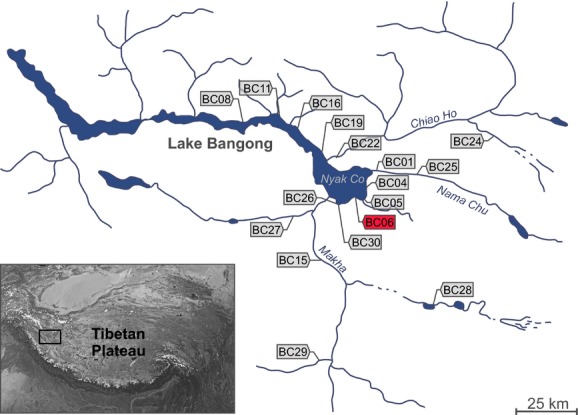
Sampling sites (with location codes) of *Gyraulus* sp. at Lake Bangong and its catchment area. Location BC06, where the aberrant (nonplanispiral) specimens were found, is highlighted in red. Detailed locality information is given in the Supporting information (see Table S1).

Lake Bangong is the largest lake of western Tibet (33.4–34.0°N, 78.4–79.9°E; 4241 m a.s.l.), covering an area of 604 km^2^ (Fontes et al. 1996). This dimictic lake is composed of five basins, which are separated by shallow sills (Hutchinson 1937; Norin 1982) and which are usually treated as individual lakes connected by rivers (see Fig.1; Fontes et al. 1996). The largest and easternmost basin is called Lake Nyak (max. depth of 41.3 m; Fontes et al. 1996). Together with the adjacent basin, it forms East Bangong. The remaining basins are called West Bangong. It is assumed that Lake Bangong is either a tectonic (Huang et al. 1989) or a moraine-dammed lake (Norin 1982), surrounded by a montane desert steppe (Fontes et al. 1996). A salinity gradient (from fresh in the east to oligosaline in the west) is observed, caused by increased evaporation along the chain of lakes (for more details, see Fontes et al. 1996; Gasse et al. 1996; Hui et al. 1996; Van Campo et al. 1996).

The Lake Bangong drainage basin (hereafter termed as Lake Bangong system) was investigated during fieldwork conducted in September 2012. Specimens of the freshwater gastropod genus *Gyraulus*, among others, were collected from 16 different sites and preserved in 80% ethanol. As mentioned before, two different shell morphotypes, planispiral and nonplanispiral (open-coiled corkscrew like morphs displaying pseudo-dextrality), were found at a single location (a pond close to Lake Bangong = locality BC06; see Fig.1). This population was further investigated for ecological (environmental), morphological, and genetic variability and compared to the other populations (see Table S1.

### Morphological data

Whole shells as well as protoconchs of three nonplanispiral (location BC06) and two planispiral specimens (BC06 and BC05) were studied by scanning electron microscopy (SEM) using a ZEISS SUPRA 40 VP Ultra (Carl Zeiss AG, Jena, Germany). Images of the remaining nonplanispiral *Gyraulus* specimens were photographed with a digital microscope system (KEYENCE VHX-2000; KEYENCE Corp., Itasca, IL, USA).

### DNA extraction, amplification, and sequencing

Specimens from 16 locations were used for genetic analyses, plus a specimen of the Yunnan Lake Lugu endemic *G. luguhuensis* (which is also known for an aberrant shell form; for details, see Shu et al. 2013). Prior to the genetic work, some corkscrew-like specimens were randomly selected, dissected, and macroscopically checked for the presence of parasites (e.g., trematodes). As a result, there was no obvious evidence for parasitism.

Genomic DNA was extracted from 37 specimens, including four specimens with nonplanispiral shells and *G*. *luguhuensis* (see Table S1), using the CTAB protocol described by Wilke et al. (2006). For phylogenetic and network analyses, we obtained sequences from two mitochondrial gene fragments: cytochrome *c* oxidase subunit I (COI, 655 bp length) and large ribosomal subunit rRNA (LSU rRNA or 16S, 414–444 bp length). The following primers for PCR (polymerase chain reaction) amplification were used: LCO1490 (Folmer et al. 1994) and COR722b (Wilke and Davis 2000) for the COI fragment and 16Sar-L and 16Sbr-H (Palumbi et al. 1991) for the 16S fragment. DNA sequencing was carried out according to the protocol described by Oheimb et al. (2013). All newly generated sequences are available from GenBank (for detailed information, see Table S1).

### Dataset composition and DNA alignment

For our study, we used the *Gyraulus* dataset published by Oheimb et al. (2013) available from GenBank (KC495714–KC495956; including four specimens from Lake Bangong system: H049–050, H052, and H055, see Table S1). In addition, 73 (37 COI, 36 16S; see Table S1) newly generated sequences were used for the reconstruction of phylogenetic relationships of the Lake Bangong system specimens. Altogether, our dataset comprised 168 *Gyraulus* specimens (168 COI and 150 16S sequences) from 51 locations on the Tibetan Plateau (including the 16 new locations in the Lake Bangong system) and the adjacent Himalayan mountain range as well as 13 locations in Asia (e.g., India and Japan), Europe (e.g., Germany and Turkey) and Africa (e.g., South Africa and Madagascar). The following three planorbid taxa were used as outgroup: *Choanomphalus maacki* (GenBank accession numbers COI/16S: EF012168/KC495843), *Hippeutis complanatus* (EF012170/KC495842), and *Planorbis planorbis* (EF012175/KC465841).

The protein-coding COI sequences were aligned by eye leaving a final alignment of 670 bp (including a 15-bp-long insertion in the outgroup taxa *C. maacki* and *P. planorbis*; see Albrecht et al. 2007). The alignment of 16S sequences was performed following the instructions of Kjer et al. (2009) for structural alignment based on the LSU rRNA structure model of *Gyraulus* spp. established by Oheimb et al. (2013). In total, two regions (positions 49–54 and 244–265) were excluded from the 16S dataset because no reliable alignment could be achieved, leaving a final alignment of 431 bp.

### Phylogenetic and network analyses

Single gene datasets were tested for substitutional saturation using the test of Xia et al. (2003) implemented in DAMBE 5.2.73 (Xia and Lemey 2009). The test showed only little saturation for both partitions even under the unlikely assumption of an asymmetrical tree. Subsequent phylogenetic analyses were conducted using all unique haplotypes of a concatenated dataset of COI and 16S (with a total of 117 haplotypes). Partition-specific best-fit substitution models were selected using jModelTest 2.1.4 (Darriba et al. 2012) based on AICc model selection (HKY + I + G for the COI fragment, F81 + G for 16S loop regions). For the stem regions of the 16S partition, the 16B model (Schöniger and von Haeseler 1994) was used. Phylogenetic relationships were reconstructed using Bayesian inference implemented in MrBayes 3.1.2 (Huelsenbeck and Ronquist 2001); settings were chosen as described in Oheimb et al. (2013): ngen = 9000000, samplefreq = 1000, and burnin = 900 (10%). The combined set of trees (two parallel runs in MrBayes) showed both high ESS (effective sample size) values (>750 for all parameters) and a smooth frequency plot as visualized in Tracer 1.5.0 (Drummond and Rambaut 2007). Finally, we used the program TreeAnnotator 1.7.5 (Drummond and Rambaut 2007) to generate a maximum clade credibility (MCC) tree (for details, see Oheimb et al. 2013).

A parsimony network analysis (connecting limit = 95%) was performed using TCS 1.21 (Clement et al. 2000) for a concatenated COI + 16S *Gyraulus* dataset sampled at the Lake Bangong system.

### Environmental data

The water parameters pH, temperature, conductivity, oxygen saturation, as well as the concentration of seven cations (potassium = K^+^, sodium = Na^+^, magnesium = Mg^2+^, calcium = Ca^2+^, strontium = Sr^2+^, iron = Fe^2+^, zinc = Zn^2+^, manganese = Mn^2+^), and hydrocarbonate (HCO^3^) were investigated for 15 of 16 locations (see Table S2; F. Wilckens, U. Wiechert, J. A. Schuessler, and M. Weynell, pers. comm.). A principle component analysis (PCA) was performed with a correlation matrix using the software Past 3.01 (Hammer et al. 2001), in order to identify possible differences among the locations (especially with respect to location BC06).

A CORONA satellite image of location BC06 and its surrounding area was purchased from the US Geological Survey (entity ID: DS1048-1134DA091; coordinates 33.480°N, 79.718°E; camera resolution: stereo medium; acquisition date: 27-SEP-1968) in order to assess temporal differences in, for example, surface area and water level, potentially affecting habitat structure compared to present-day conditions.

## Results

### Shell morphology

**Figure 2:**
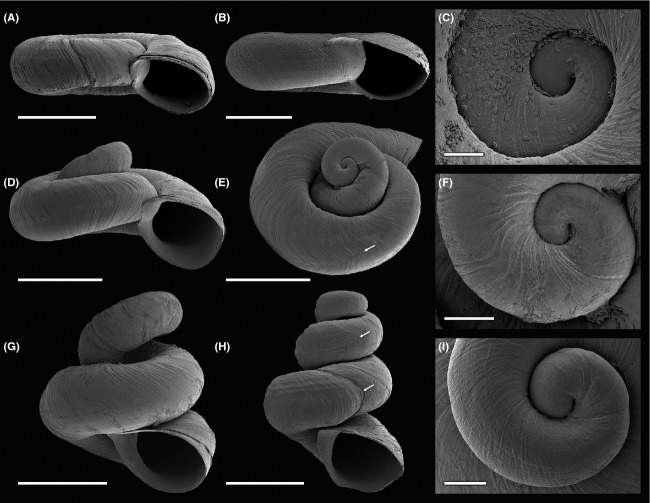
SEM images of shells (planispiral and nonplanispiral) from extant *Gyraulus* sp. caught alive in the Lake Bangong drainage system. (A) Most abundant morph in the pond (location BC06, see Fig.1), which shows slight shell distortion; scale bar = 1.5 mm. (B–C) Typical planispiral morph of eastern Lake Nyak (location BC05). (B) Teleoconch; scale bar = 1.5 mm. (C) Early ontogenetic shell with *Cocconeis* sp. diatoms attached and protoconch with characteristic lirae; scale bar = 0.1 mm. (D–F) Nonplanispiral morph (intermediate form) with strong shell distortion between second and third whorl. (D–E) Teleoconch; scale bars = 1.5 mm. (F) Early ontogenetic shell with protoconch; scale bar = 0.2 mm. (G–I) Nonplanispiral (open-coiled scalariform) morphs. (G–H) Teleoconch; scale bars = 1.5 mm. (I) Early ontogenetic shell with protoconch; scale bar = 0.2 mm. Significant structural shell irregularities are highlighted with white arrows.

Altogether, 29 *Gyraulus* specimens were collected at the only location with the aberrant shell form (BC06). Six specimens (frequency of 20.7%) showed a clearly aberrant nonplanispiral (corkscrew-like) morphotype under the stereo microscope, three had a more intermediate form (frequency of 10.3%; see Fig. S2). The remaining 20 specimens (frequency of 69.0%) belonged to the typical planispiral morphotype. There was no obvious difference in shell width. However, shell height was more than twice in the aberrant morphs (see Fig.2 and Fig. S2). Moreover, the nonplanispiral specimens exhibited an open-coiled and pseudo-dextral shell, forming a corkscrew-like habitus.

However, under the SEM, it could be observed that specimens that appeared to be planispiral under the stereo microscope were actually slightly (pseudo-) dextral (Fig.2A), and growth increments were more pronounced compared to specimens from Lake Nyak (Fig.2B). Moreover, the distortion of shell from planispiral to slightly dextral (Fig.2A) or corkscrew-like (e.g., Fig.2H) morphs started with the first teleoconch whorl. Diatoms of the alkalibiontic genus *Cocconeis* were commonly attached to specimens from Lake Nyak (Fig.2C), but in low numbers or not at all to specimens from location BC06. The protoconch had a diameter of 0.4–0.5 mm and exhibited lirae. In some specimens, the lirae were less pronounced and the growth of the embryonic shell showed irregularities (Fig.2I). The teleoconch of most specimens from BC06 exhibited growth irregularities such as V-shaped increments (Fig.2E) or an offset of shell growth (Fig.2H).

### Phylogenetic and network analyses

**Figure 3:**
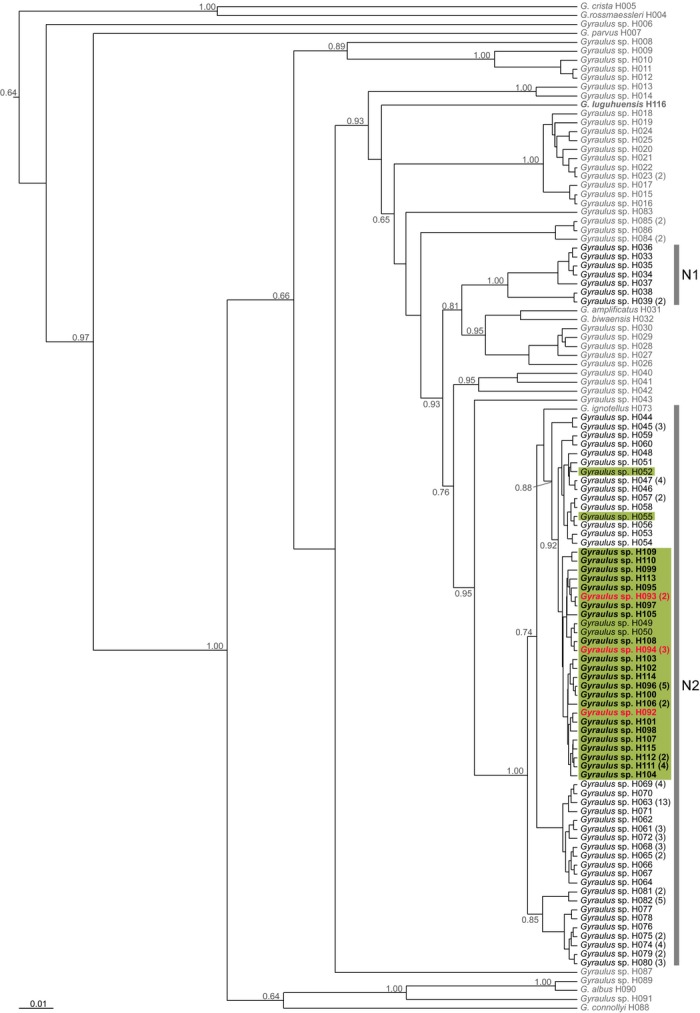
Maximum clade credibility (MCC) tree of *Gyraulus* spp. (resulting from the MrBayes analysis) based on the concatenated dataset of COI and 16S. Specimens are labeled with haplotype codes adopted from Oheimb et al. (2013) and extended by the newly sequenced specimens (H092–H116; in bold; for details, see Table S1). Tibetan Plateau haplotypes are marked in black and nonplanispiral specimens from the Tibetan Plateau in red; Lake Bangong haplotypes are highlighted with green rectangles. The Tibetan Plateau *Gyraulus* clades [N1 and N2, N = north of Himalayan mountain range; adopted from Oheimb et al. (2013)] are marked with gray bars. The outgroup taxa *Planorbis planorbis*, *Choanomphalus maacki*, and *Hippeutis complanatus* were removed from the tree a posteriori. Bayesian posterior probabilities (BPP) are given for deeper nodes (when BPP ≥0.5). The scale bar represents substitutions per side according to the applied model of sequence evolution.

The phylogenetic reconstruction of the combined dataset (COI and 16S) resulted in a topology that is consistent with the *Gyraulus* phylogeny of Oheimb et al. (2013). Therefore, names of clades and haplotypes were adopted from the latter source (Fig.3). Both phylogenetic analyses (Oheimb et al. 2013 and this study) showed that the Tibetan Plateau specimens belong to two clades (N1 and N2, N = north of Himalayan mountain range; see Fig.3) supported by a Bayesian posterior probability (BPP) value of 0.93. Clade N1 includes haplotypes H033 to H039 and is sister to *G*. *amplificatus* and *G*. *biwaensis* from Japan and *Gyraulus* sp. from Montenegro (H028) and China (H026–027, H029–030). Clade N2 consists of haplotypes H044 to H072, H074 to H082, H092 to H115, and the Russian *G*. *ignotellus* (H073) and is sister to *Gyraulus* sp. from Nepal (H043). Notably, both planispiral and nonplanispiral specimens from the Lake Bangong system (H049–050, H092–115), except for H052 and H055 form a well-supported group within clade N2 (BPP = 0.92). The phylogenetic relationship of the newly sequenced Lake Lugu endemic *G*. *luguhuensis* remains unclear (low node support of BPP <0.5), probably due to poor sampling (only one individual).

**Figure 4:**
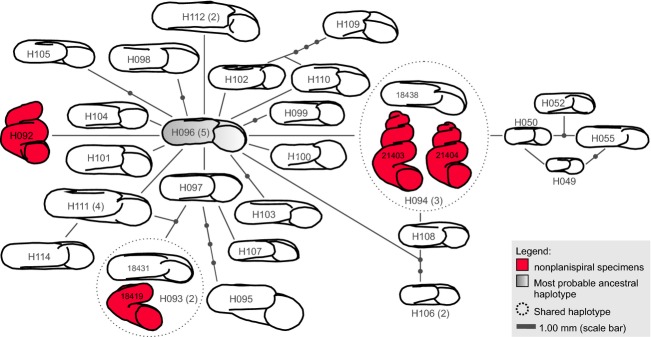
Statistical parsimony network (connecting limit: 95%) based on the concatenated dataset of COI and 16S of the Lake Bangong *Gyraulus* clade (including 26 unique haplotypes: H049–H050, H092–H115). Only one shell shape is illustrated if identical haplotypes show identical shell types (planispiral or nonplanispiral).

The parsimony network analysis of the concatenated dataset for the monophyletic Lake Bangong system clade (including 38 *Gyraulus* specimens; see Fig.3) resulted in a single network with 26 haplotypes separated from each other by max. 11 mutational steps (see Fig.4). Haplotype H096 has the highest probability to be ancestral and is shared by five specimens (see Table S1). The haplotypes of the nonplanispiral specimens (found in haplotypes H092–H094) do not form a single cluster. Interestingly, haplotypes H094 and H093 are shared by planispiral and nonplanispiral specimens.

### Environmental data

**Figure 5:**
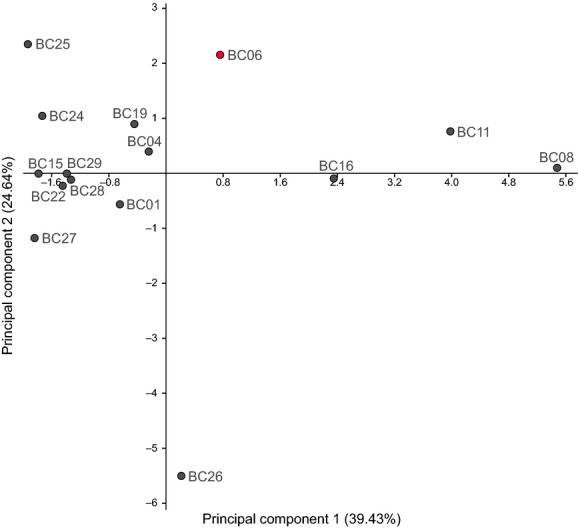
Scatter plot of the principal component analysis (PCA) based on 14 different environmental parameters among the sampling sites of *Gyraulus* sp. at Lake Bangong and its catchment area. Location BC06, where the aberrant (nonplanispiral) *Gyraulus* specimens were found, is highlighted in red. Detailed locality information plus the respective parameters are given in the Supplementary material (see Tables S1 and S2).

The differentiation of the Lake Bangong system locations based on their composition of physical and chemical parameters is visualized by the PCA (Fig.5). The first two axes of the PCA accounted together for 66.4% of the total variation (axis 1: for 39.43%, eigenvalue = 5.1; axis 2: 24.64%, eigenvalue = 3.2). Thus, locations clustering closely together are more similar regarding their physical and chemical parameters. Moreover, the plot shows that location BC06 is distinct from the “main cloud” of locations (but not as clearly separated as location BC26). The distinctiveness of BC06 might be due to, for instance, an elevated pH value of 10.4 as compared to the mean pH of 8.7 (see Table S2 for water parameters).

In 1968 (see Fig. S2), the pond was clearly separated from Lake Nyak by a 40–70 m stretch of land. It had a maximum length of 90 m and a maximum width of 50 m. The catchment area of the pond was located south of the pond and covered a distance of ca. 0.5 km to the slope of a mountain. At higher elevations around the pond as well as in the Lake Nyak basin, palaeo-shorelines were visible (arrows in Fig. S2). A road track ran along the shore of Lake Nyak and thus passed the pond. In contrast, the water level of the pond (BC06) as well as of Lake Nyak was significantly higher in 2012. The maximum length of the pond was 140 m and the maximum width about 90 m, and the surface area was approximately 0.05 km^2^. The narrow portion of land separating the pond from Lake Nyak – only 6–7 m wide compared to 40 m in 1968, and only about 0.6–0.8 m above water level – had been formed as a beach wall but was overprinted by repeated repairing of the road track which run along the lake. Water levels of the pond and Lake Nyak were the same, indicating transgression of lake water into the pond. The pond is also periodically fed by a single stream.

## Discussion

### Phylogenetic status of the *Gyraulus* populations and history of the Lake Bangong system

All newly sequenced *Gyraulus* specimens from the Lake Bangong system, including the aberrant nonplanispiral specimens, clustered within the endemic Tibetan Plateau clade N2. Remarkably, the present phylogenetic analysis of *Gyraulus* spp. is fully consistent with the paper of Oheimb et al. (2013). Both studies demonstrate that the high-elevation Tibetan Plateau has been colonized by *Gyraulus* at least two times independently (Tibetan Plateau clades: N1 and N2; see Fig.3). This is also true for the extant Lake Bangong system. Interestingly, multiple colonization of this system is also known for sphaeriid bivalves (at least six independent colonizations; Clewing et al. 2013). In addition, the system is also home to one of the few Tibetan populations of the nonpulmonate gastropod *Valvata* sp. (Clewing et al. 2014). Altogether, Lake Bangong potentially harbors the highest mollusk diversity currently recognized throughout the high-elevation Tibetan Plateau. This high diversity might be related to the geographic position of the lake system at the western margin of the plateau (Upper Indus freshwater ecoregion, Abell et al. 2008) and thus close to other freshwater ecoregions compared to central Tibetan Plateau lakes. Another possible explanation for the high mollusk diversity of Lake Bangong as well as for the appearance of extraordinary shell forms might be the comparatively old age of the lake system, which probably has existed since at least the LGM (e.g., Oheimb et al. 2011).

The palaeo-shorelines observed at Lake Bangong (see Fig. S2) indicate former higher lake levels, which were probably reached last during the Holocene Climate Optimum (ca. 10–7 ka BP) (Fontes et al. 1996; Gasse et al. 1996). Moreover, Hutchinson (1937) suggested that the lake level again increased in the Lake Bangong Co system during the late 19th century, although it remains unclear when the pond has exactly developed and how frequent exchanges with Lake Nyak occurred. Nonetheless, lake-level fluctuations directly affect the water chemistry of surrounding water bodies, possibly resulting in unstable habitats and/or environmental stress for freshwater organisms. Prior to the most recent lake-level rise in the 19th century, the salinity in this closed-basin pond (pond at location BC06) is assumed to have been too high for mollusks (*Gyraulus* spp. can only tolerate up to 5.2 psu; Gittenberger et al. 1998). It is thus concluded that *Gyraulus* sp. likely dispersed into the pond from Lake Bangong only a few decades ago, suggesting a relative young timeframe for the evolution of the aberrant shell forms.

### Morphological variation and correlation with environmental variables

Shell shape in gastropods can be generally described by four basic parameters: the shape of the generating curve, the whorl expansion rate, the position of the generating curve in relation to the axis, and the rate of whorl translation (for details, see Raup 1966). Starting with a typical planispiral *Gyraulus* shell, the increase in the rate of translation will result in nonplanispiral or, in an extreme case, corkscrew-like shells (detached whorls) as illustrated for some Lake Bangong specimens.

There are two major molecular-based mechanisms that might be responsible for these shell aberrations, variation caused by evolutionary factors (e.g., mutations and natural selection), and ecophenotypic plasticity (or potentially a combination of both). In the first scenario, we would expect a link between molecular and morphological patterns, for example, that nonplanispiral Tibetan Plateau specimens would cluster together or even constitute a monophyletic group. If so, the non-random mating of nonplanispiral and planispiral specimens (i.e., assortative mating) might play an important role. In the second scenario, we would not necessarily expect to see a concordance between both patterns due to the often nonheritable characteristic of ecophenotypic plasticity.

Our results support the scenario that ecophenotypic plasticity may have led to the observed shell variability given the present phylogenetic pattern of the corkscrew-like specimens (e.g., nonmonophyletic group; see section above). Ecophenotypic modifications represent a possible response of a phenotype to habitat heterogeneity (Schlichting and Pigliucci 1998; Pigliucci 2001; Pfennig et al. 2010), for example, changes in water chemistry (Arter 1989; Dunithan et al. 2012). Thus, growth irregularities shown for the embryonic shell of *Gyraulus* specimens from location BC06 (see Fig.2) might be the result of altered environmental parameters influencing the embryos that develop over several weeks in egg capsules embedded in a gelatinous mass (Riedel 1993).

Interestingly, ecophenotypic modifications are also considered to be responsible for some of the aberrant shell morphs observed within the highly diverse fossil Steinheim *Gyraulus* fauna, particularly in *G*. *denudatus* and *G*. *triquetrus* (Reif 1983; Rasser 2013; see Fig. S3). These species show striking similarities with the nonplanispiral specimens from the Tibetan Plateau. For the Steinheim *Gyraulus* fauna, it is assumed that environmental pressure caused an increase in shell variability (e.g., altered water chemistry due to a higher evaporation rate; Bajor 1965; Tütken et al. 2006). Note, however, that ecophenotypic modifications are considered to explain only part of the Steinheim *Gyraulus* morphospace. Most of the morphs are currently regarded as biospecies because of their long-term occurrence throughout the fossil record (Rasser 2013).

Remarkably, the PCA (Fig.5) does not indicate that environmental variables measured at locality BC06 strongly differ from other localities in the Lake Bangong system. The only parameter potentially deviating from regular conditions is the pH of 10.4. This value is 0.8 higher than the maximum values published for habitats occupied by *Gyraulus* (Økland 1990). However, the latter study was conducted in Norway, where waters tend to be acidic. Given the information at hand, we have to assume that there are other possible explanations for the observed pattern. In fact, Kitching et al. (1966) could show that wave and predation intensity can lead to changes in foot size as well as shell morphology in the intertidal marine snail of the genus *Nucella*. Similar effects of predator pressure on phenotypic variability were also observed in freshwater organisms such as bivalves (Inoue et al. 2013). Interestingly, a recent study of Rasser and Covich (2014) on *Gyraulus* shells from Lake Steinheim led to the assumption that variation in shell thickness and morphology was triggered by predation pressure (defense against shell-breaking predators). However, typical molluskivore predators such as fishes, leeches, crabs, and predatory insects (e.g., dragonflies, water beetles) could not be observed for the location where the corkscrew-like *Gyraulus* specimens were found and thus predatory pressure might not be the most likely cause for the observed shell variability. This is also supported by the observation that some embryonic shells show irregular growth – a phenomenon not relatable to predation. The V-shaped growth increments of Lake Bangong system *Gyraulus* (see Fig.2), which are probably caused by arthropod predators, however, can be found in both regular and aberrant shells.

Another important issue in the context of ecophenotypic plasticity is the possible effect of parasites on the morphology of its host. It is well documented that the infection of snails by trematode parasites can either lead to alterations of shell shapes (Krist 2000; McCarthy et al. 2003) or might enhance shell growth resulting in abnormally large sizes (i.e., gigantism; Sousa 1983; Sorensen and Minchella 2001). However, macroparasites could not be observed in the aberrant Tibetan Plateau *Gyraulus* specimens, while an effect of microparasites (not analyzed in this study) cannot be entirely excluded as trigger for the observed aberrant shell forms. Given these observations, it is assumed that the nonplanispiral shell shapes are not controlled by biotic interaction.

### Extraordinary shell forms in extant freshwater gastropods

There are several studies about aberrant shell forms (open-coiled corkscrew-like morphs) in fossil freshwater gastropods (e.g., Nützel and Bandel 1993; Harzhauser et al. 2002; Wesselingh 2006; Scholz and Glaubrecht 2010). However, only few are related to extant freshwater faunas. Interestingly, some of the recent aberrant shell morphs have been described as distinct endemic species, for example, *Heleobia mirum* (Lake Titicaca), *Liobaicalia stiedae* (Lake Baikal), and *Gocea ohridana* (Lake Ohrid). Other morphs are interpreted as sporadical phenomena within a species of otherwise regular shell morphology, for example, in *Anisus leucostoma*, *Lymnaea stagnalis*, *Biomphalaria glabrata,* and *Valvata lewisi* morph *ontariensis* (for more details, see Table1). Note that in some studies, it remains uncertain whether the observed morphs represent valid species or whether they are the result of hybridization, incomplete lineage sorting, ecophenotypic plasticity, or confused taxonomy (e.g., *H. mirum*; Kroll et al. 2012).

Interestingly, *H. mirum*, *L. stiedae*, and *G. ohridana*, which always exhibit corkscrew-like shells, all belong to the Caenogastropoda and are endemic to rather stable ancient lake systems (see Table1). The remaining recent examples belong to the group of Heterobranchia and are mainly recorded from unstable habitats. The peripheral pond with the nonplanispiral specimens may represent such an unstable environment as the CORONA satellite image shows significantly smaller surface area of the pond in 1968 (see Fig. S2). Remarkably, *A. leucostoma* and *B. glabrata* only developed aberrant shell forms (within the next generations) when living specimens were transferred from the natural habitat to the laboratory (Boettger 1949; Basch 1968). Both observations suggest that the alteration of environmental conditions (e.g., pH) can lead to the development of aberrant shell morphs.

However, specimens with corkscrew-like (open-coiled) shells appear to have clear disadvantages compared to those with coiled shells. Open-coiling might have a high impact on the mobility (e.g., reduced speed) of the respective individuals as illustrated for the open-coiled morphs of *V. lewisi* by Clarke (1973). Additionally, they encounter difficulties of balancing the shell and surface friction (Rex and Boss 1976) and are potentially subjected to a higher pressure of molluskivorous predators (e.g., fishes, crabs). This might also be the reason why corkscrew-like shell shapes, in general, are extremely rare among freshwater gastropods. The aberrant Lake Bangong system *Gyraulus* specimens are, to our knowledge, probably only the third record (see Table1) of such morphs within the family Planorbidae. Assuming that, on the one hand, a high percentage of these morphs result from ecophenotypic plasticity (nonheritable characteristic) and that, on the other hand, records from natural populations remain limited, it might be possible that the general frequency of the phenomenon is underestimated (e.g., due to a sampling artifact).

## Concluding Remarks

Our study represents the first evolutionary analysis of living corkscrew-like *Gyraulus* specimens – collected from the high-elevation Tibetan Plateau. It thus provides an important recent analogue to the Steinheim Basin and a prime model system for the examination of extraordinary shell shape evolution. Given the potential ephemerality of the phenomenon, the compact phylogeographical network patterns inferred, the likely young phylogenetic age of the aberrant *Gyraulus* shells studied, and the ecological peculiarities of the study site, we find no supporting evidence that the corkscrew-like shell is genetically fixed nor that it evolved over an extended period of time.

Instead, the results indicate that the evolution of the aberrant shell forms on the Tibetan Plateau could likely be considered as a rapid ecophenotypic response, possibly induced by ecological stress. This finding may thus have important implications for the ongoing debate about the processes that have caused the extraordinary shell diversity in palaeolakes such as the Steinheim Basin.

However, it remains uncertain which specific processes and underlying factors are, indeed, responsible for the sporadic appearance of such corkscrew-like shells in both the fossil and extant freshwater gastropod records. Hence, further studies including new material should focus on breading experiments (test of heritability of the shell form), mating experiments (test of assortative mating), and transcriptomics (gene expression analyses of functional genes) in order to elucidate the basic mechanism behind the evolution of aberrant shell forms.

## Supporting Information

**Table S1.** List of studied specimens including taxon, haplotype number [adopted from Oheimb et al. (2013) and extended by the newly sequenced *Gyraulus* specimens: H092–H116], DNA voucher number, shell type (planispiral or nonplanispiral), locality information (including locality codes for all sites illustrated in Fig. 1), and GenBank accession numbers. **Table S2.** List of locations and their particular environmental parameters used for principle component analysis (see Fig. 5; F. Wilckens, U. Wiechert, J. A. Schuessler, and M. Weynell, pers. comm.). **Figure S1.** Shell images of all nonplanispiral *Gyraulus* specimens sampled at location BC06 located within the Lake Bangong system. **Figure S2.** CORONA satellite photograph of the south-eastern shore of the easternmost lake basin (Nyak Co) of the Lake Bangong system taken in 1968. **Figure S3.** SEM images of fossilized *Gyraulus* specimens showing corkscrew-like (open-coiled) shells found in the Miocene Steinheim Basin (Germany).
