# Supplementary material for: Ecophenotypic plasticity leads to extraordinary gastropod shells found on the “Roof of the World”
Source: Ecol Evol. 2015 Jul 3;5(14):2966–79. doi: 10.1002/ece3.1586 (PMC4541999; doi:10.1002/ece3.1586)
Supplement: Supplementary file 1 [file ece30005-2966-sd1.docx]

**Supporting Information**

**Table S1.** List of studied specimens including taxon, haplotype number [adopted from Oheimb et al. (2013) and extended by the newly sequenced *Gyraulus* specimens: H092–H116], DNA voucher number, shell type (planispiral or nonplanispiral), locality information (including locality codes for all sites illustrated in Fig. 1), and GenBank accession numbers. *Sequences obtained from GenBank (source: Oheimb et al. 2013; sampled 2009). Location BC06, where the aberrant (nonplanispiral) *Gyraulus* specimens were found, is highlighted in red.

| **Taxon** | **Haplotype no.** | **DNA voucher no.** | **Shell shape**  **type** | **Locality code** | **Locality (China)** | **Coordinates** | **COI GenBank accession no.** | **16S GenBank accession no.** |
| --- | --- | --- | --- | --- | --- | --- | --- | --- |
| *Gyraulus* sp. | H049* | 16124 | planispiral | BC30 | Tibet, Lake Bangong (close to BC26) | 33.44151°N, 79.78342°E | KC495816 | KC495935 |
|  | H050* | 16123 | planispiral | BC30 | Tibet, Lake Bangong (close to BC26) | 33.44151°N, 79.78342°E | KC495815 | KC495934 |
|  | H052* | 15028 | planispiral | BC30 | Tibet, Lake Bangong (close to BC26) | 33.44151°N, 79.78342°E | KC495740 | KC495868 |
|  | H055* | 16125 | planispiral | BC30 | Tibet, Lake Bangong (close to BC26) | 33.44151°N, 79.78342°E | KC495817 | KC495936 |
|  | H092 | 18418 | nonplanispiral | BC06 | Tibet, pond near Lake Bangong | 33.47221°N, 79.85165°E | KR816730 | KR816767 |
|  | H093 | 18419 | nonplanispiral | BC06 | Tibet, pond near Lake Bangong | 33.47221°N, 79.85165°E | KR816731 | KR816768 |
|  | H093 | 18431 | planispiral | BC01 | Tibet, inflow (Nama Chu) of Lake Bangong | 33.56812°N, 79,94763°E | KR816737 |  |
|  | H094 | 21403 | nonplanispiral | BC06 | Tibet, pond near Lake Bangong | 33.47221°N, 79.85165°E | KR816764 | KR816800 |
|  | H094 | 21404 | nonplanispiral | BC06 | Tibet, pond near Lake Bangong | 33.47221°N, 79.85165°E | KR816765 | KR816801 |
|  | H094 | 18438 | planispiral | BC19 | Tibet, northern shore of Lake Bangong | 33.63363°N, 79.71366°E | KR816740 | KR816776 |
|  | H095 | 18420 | planispiral | BC06 | Tibet, pond near Lake Bangong | 33.47221°N, 79.85165°E | KR816732 | KR816769 |
|  | H096 | 18421 | planispiral | BC06 | Tibet, pond near Lake Bangong | 33.47221°N, 79.85165°E | KR816733 | KR816770 |
|  | H096 | 18434 | planispiral | BC28 | Tibet, small lake south-east of Lake Bangong | 33.10310°N, 80.19607°E | KR816738 | KR816774 |
|  | H096 | 21385 | planispiral | BC08 | Tibet, northern shore of Lake Bangong | 33.72546°N, 79.39147°E | KR816746 | KR816782 |
|  | H096 | 21381 | planispiral | BC15 | Tibet, sidearm of inflow (Makha) of Lake Bangong | 33.23835°N, 79.78357°E | KR816742 | KR816778 |
|  | H096 | 21397 | planispiral | BC04 | Tibet, eastern shore of Lake Bangong | 33.51515°N, 79.90436°E | KR816758 | KR816794 |
|  | H097 | 18426 | planispiral | BC05 | Tibet, eastern shore of Lake Bangong | 33.46476°N, 79.87551°E | KR816734 | KR816771 |
|  | H098 | 18427 | planispiral | BC05 | Tibet, eastern shore of Lake Bangong | 33.46476°N, 79.87551°E | KR816735 | KR816772 |
|  | H099 | 18430 | planispiral | BC01 | Tibet, inflow (Nama Chu) of Lake Bangong | 33.56812°N, 79,94763°E | KR816736 | KR816773 |
|  | H100 | 18435 | planispiral | BC28 | Tibet, small lake south-east of Lake Bangong | 33.10310°N, 80.19607°E | KR816739 | KR816775 |
|  | H101 | 18439 | planispiral | BC19 | Tibet, northern shore of Lake Bangong | 33.63363°N, 79.71366°E | KR816741 | KR816777 |
| Continued**.** |  |  |  |  |  |  |  |  |
| **Taxon** | **Haplotype no.** | **DNA voucher no.** | **Shell shape**  **type** | **Locality code** | **Locality (China)** | **Coordinates** | **COI GenBank accession no.** | **16S GenBank accession no.** |
|  | H102 | 21382 | planispiral | BC15 | Tibet, sidearm of inflow (Makha) of Lake Bangong | 33.23835°N, 79.78357°E | KR816743 | KR816779 |
|  | H103 | 21383 | planispiral | BC26 | Tibet, wetlands south of Lake Bangong | 33.44201°N, 79.78291°E | KR816744 | KR816780 |
|  | H104 | 21384 | planispiral | BC26 | Tibet, wetlands south of Lake Bangong | 33.44201°N, 79.78291°E | KR816745 | KR816781 |
|  | H105 | 21386 | planispiral | BC08 | Tibet, northern shore of Lake Bangong | 33.72546°N, 79.39147°E | KR816747 | KR816783 |
|  | H106 | 21387 | planispiral | BC11 | Tibet, northern shore of Lake Bangong | 33.75324°N, 79.54513°E | KR816748 | KR816784 |
|  | H106 | 21388 | planispiral | BC11 | Tibet, northern shore of Lake Bangong | 33.75324°N, 79.54513°E | KR816749 | KR816785 |
|  | H107 | 21389 | planispiral | BC16 | Tibet, northern shore of Lake Bangong | 33.69326°N, 79.61760°E | KR816750 | KR816786 |
|  | H108 | 21390 | planispiral | BC16 | Tibet, northern shore of Lake Bangong | 33.69326°N, 79.61760°E | KR816751 | KR816787 |
|  | H109 | 21391 | planispiral | BC22 | Tibet, sidearm of northern inflow of Lake Bangong | 33.62733°N, 79.76977°E | KR816752 | KR816788 |
|  | H110 | 21392 | planispiral | BC22 | Tibet, sidearm of northern inflow of Lake Bangong | 33.62733°N, 79.76977°E | KR816753 | KR816789 |
|  | H111 | 21393 | planispiral | BC24 | Tibet, sidearm of inflow of Lake Bangong | 33.66933°N, 80.46586°E | KR816754 | KR816790 |
|  | H111 | 21394 | planispiral | BC24 | Tibet, sidearm of inflow of Lake Bangong | 33.66933°N, 80.46586°E | KR816755 | KR816791 |
|  | H111 | 21399 | planispiral | BC27 | Tibet, southern inflow to Lake Bangong | 33.41452°N, 79.64413°E | KR816760 | KR816796 |
|  | H111 | 21400 | planispiral | BC27 | Tibet, southern inflow to Lake Bangong | 33.41452°N, 79.64413°E | KR816761 | KR816797 |
|  | H112 | 21395 | planispiral | BC25 | Tibet, eastern inflow to Lake Bangong | 33.55812°N, 80.05582°E | KR816756 | KR816792 |
|  | H112 | 21396 | planispiral | BC25 | Tibet, eastern inflow to Lake Bangong | 33.55812°N, 80.05582°E | KR816757 | KR816793 |
|  | H113 | 21398 | planispiral | BC04 | Tibet, eastern shore of Lake Bangong | 33.51515°N, 79.90436°E | KR816759 | KR816795 |
|  | H114 | 21401 | planispiral | BC29 | Tibet, sidearm of southern inflow of Lake Bangong | 32.90556°N, 79.73295°E | KR816762 | KR816798 |
|  | H115 | 21402 | planispiral | BC29 | Tibet, sidearm of southern inflow of Lake Bangong | 32.90556°N, 79.73295°E | KR816763 | KR816799 |
| *G. luguhuensis* | H116 | 17773 | nonplanispiral | LL01 | Yunnan/Sichuan, Lake Lugu | 27.68768°N 100.78705°E | KR816766 | KR816802 |

**Table S2.** List of locations and their particular environmental parameters used for principle component analysis (see Fig. 2; F. Wilckens, U. Wiechert, J. A. Schuessler, and M. Weynell, pers. comm.). Note that BC05 is not included due to the lack of all parameters. Location BC06, where the aberrant (nonplanispiral) *Gyraulus* specimens were found, is highlighted in red. *Detailed locality information are given in Table S1.

| **Location code*** | **Environmental variables** | | | | | | | | | | | | | |
| --- | --- | --- | --- | --- | --- | --- | --- | --- | --- | --- | --- | --- | --- | --- |
|  | Water temp. (°C) | pH | Cond.  (µS/cm) | Oxyg.  (mg/l) | K^+^  (µmol/l) | Na^+^  (µmol/l) | Mg^2+^  (µmol/l) | Ca^2+^  (µmol/l) | Sr^2+^  (µmol/l) | Fe^2+^  (µmol/l) | Zn^2+^  (µmol/l) | Mn  (µmol/l) | HCO_3_^-^  (µmol/l) |  |
| BC01 | 12.6 | 8.8 | 855 | 4.30 | 140.7 | 4,045.3 | 2,057.2 | 636.3 | 12.098 | 0.36 | 0.00 | 0.16 | 4,800 |  |
| BC04 | 18.2 | 8.9 | 920 | 6.22 | 222.5 | 4,654.2 | 2,217.7 | 481.6 | 2.283 | 0.00 | 0.00 | 0.00 | 6,100 |  |
| BC06 | 19.1 | 10.4 | 2,370 | 7.50 | 529.4 | 14,745.7 | 5,184.1 | 1,003.0 | 13.696 | 0.00 | 0.00 | 0.00 | 3,500 |  |
| BC08 | 13.9 | 9.1 | 4,860 | 6.02 | 1,501.3 | 37,147.8 | 7,940.8 | 192.1 | 0.685 | 0.00 | 0.00 | 0.04 | 16,700 |  |
| BC11 | 18.8 | 9.0 | 3,930 | 7.00 | 1,176.5 | 32,623.2 | 6,500.7 | 573.9 | 2.967 | 0.00 | 0.05 | 0.09 | 16,800 |  |
| BC15 | 18.6 | 7.5 | 423 | 8.82 | 69.1 | 1,174.4 | 604.8 | 773.5 | 2.739 | 0.90 | 0.00 | 0.18 | 3,400 |  |
| BC16 | 12.9 | 9.1 | 2,650 | 6.14 | 767.3 | 19,182.4 | 4,855.0 | 274.5 | 1.027 | 0.00 | 0.00 | 0.00 | 10,700 |  |
| BC19 | 17.1 | 9.0 | 1,058 | - | 547.3 | 4,741.2 | 2,427.5 | 1,110.3 | 11.641 | 0.00 | 0.00 | 0.07 | 6,200 |  |
| BC22 | 14.8 | 7.6 | 645 | 4.92 | 465.5 | 1,805.1 | 979.2 | 1,646.8 | 11.527 | 0.00 | 0.00 | 0.04 | 5,800 |  |
| BC24 | 17.9 | 8.8 | 287 | 9.24 | 23.0 | 435.0 | 576.0 | 840.9 | 2.739 | 0.00 | 0.00 | 0.00 | 3,300 |  |
| BC25 | 23.3 | 8.4 | 731 | 10.28 | 112.5 | 2,540.3 | 1,398.9 | 1,347.4 | 18.831 | 0.00 | 0.00 | 0.00 | 5,800 |  |
| BC26 | 7.7 | 7.2 | 1,427 | 4.68 | 327.4 | 5,393.7 | 2,970.6 | 818.4 | 5.250 | 4.66 | 0.00 | 2.55 | 12,250 |  |
| BC27 | 9.8 | 8.2 | 172 | 6.43 | 40.9 | 326.2 | 207.4 | 568.9 | 1.484 | 0.00 | 0.31 | 0.05 | 1,900 |  |
| BC28 | 11.2 | 9.6 | 141 | 6.72 | 33.2 | 221.8 | 218.1 | 444.1 | 1.141 | 0.07 | 0.00 | 0.07 | 1,650 |  |
| BC29 | - | 8.9 | 307 | - | 69.1 | 856.9 | 567.8 | 716.1 | 2.168 | 0.54 | 0.00 | 0.05 | 3,200 |  |


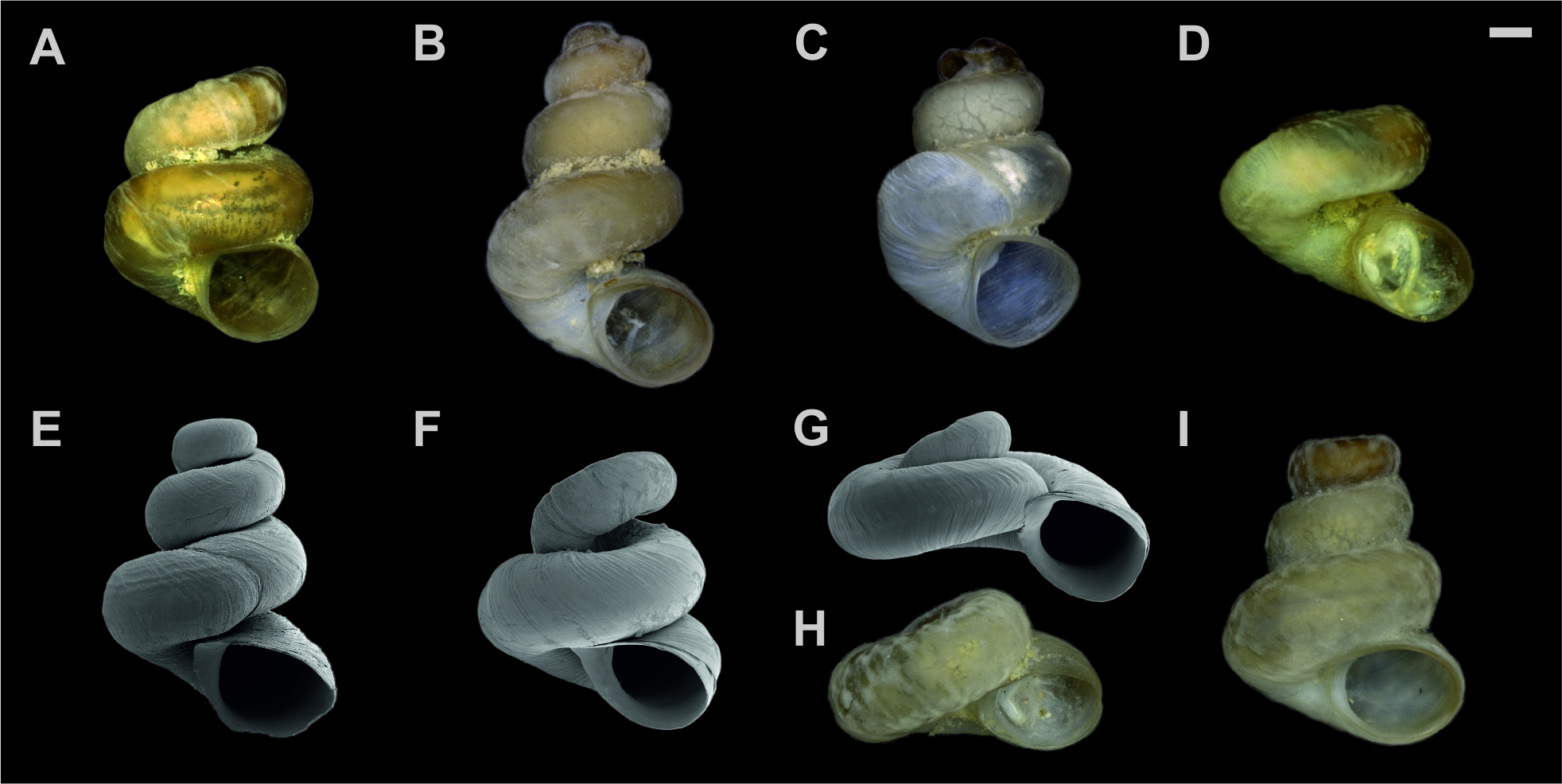


**Figure S1.** Shell images of all nonplanispiral *Gyraulus* specimens sampled at location BC06 located within the Lake Bangong system. (A–D) Specimens used for genetic analyses (haplotype no./DNA voucher no.); A: H092/18418, B: H094/21403, C: H094/21404, and D: H093/18419. (E–G) Specimens studied by scanning electron microscopy (SEM; see Fig. 3 D–I). (H–I) Other material, stored as voucher at the Systematics and Biodiversity collection of the University of Giessen (UGSB), Germany. Scale bar = 0.5 mm.


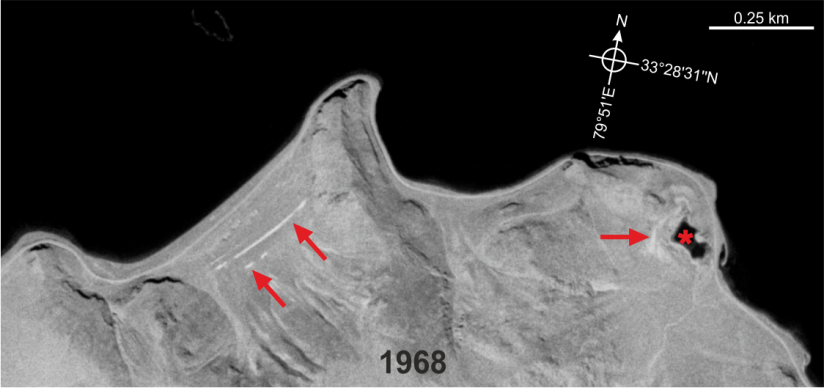


**Figure S2.** CORONA satellite photograph of the south-eastern shore of the easternmost lake basin (Nyak Co) of the Lake Bangong system taken in 1968. Palaeo-shorelines (red arrows) of higher lake levels are pronounced in the basin of the pond (red star) and in the basin of Nyak Co.


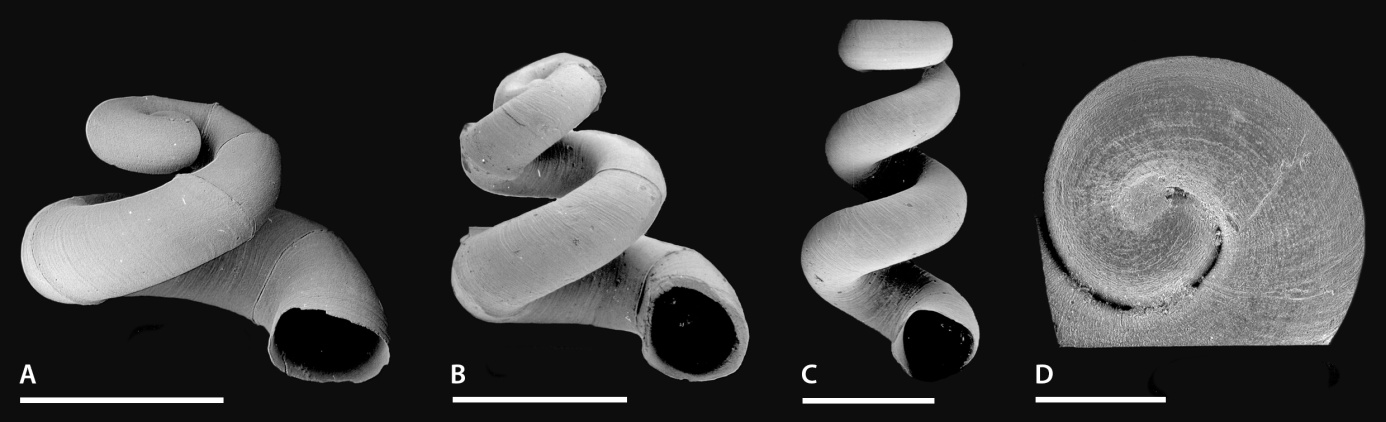


**Figure S3.** SEM images of fossilized *Gyraulus* specimens showing corkscrew-like (open-coiled) shells found in the Miocene Steinheim Basin (Germany). (A–B) Two specimens of *G. distortus*; scale bars = 0.6 mm. (C–D) *G. denudatus*; C: whole habitus; scale bar = 0.6 mm. D: protoconch; scale bar = 0.15 mm. The images illustrated here were adopted from the study of Nützel and Bandel (1993), corresponding to plate 7, figs. 4, 5a, 6a, and 6c.
